# Ventilation and Contagion

**Published:** 1869-03

**Authors:** 


### ARTICLE VIII.

### *Ventilation and Contagion.*

Dr. Wm. T. Thomas, of New York, has furnished a very accurate and thoughtful article in the Transactions of the New York Medical Society.

In 1863 there were 15,369 tenement houses in New York, with a population of about 500,000. There are only two diseases in which the mortality in New York and London are nearly equal, *viz.*, Small-Pox and Remittent Fever. Of the former disease, 1 in 1384 of the population die in London; and 1 in 1303 in New York. Of remittent fever, 1 in 32, 954 of the population in London, and 1 in 34,615 in New York.

New York has a less mortality than London, in measles, scarlet fever, quinsy, whooping-cough, erysipelas, carbuncle, influenza, rheumatism, zymotic diseases generally. Thus, 1 in 1772 of population die of measles in London, and only 1

---

in 4186 in New York. As many as 1 in 585 of population die of scarlet fever in London, and only 1 in 4186 in New York. 1 in 35,802 of quinsy, to 1 in 81,818; 1 in 1333 of whooping-cough, to 1 in 7887; 1 in 6561 of erysipelas, to 1 in 7258; 1 in 51,786 of carbuncle, to 1 in 250,000; 1 in 70,773 of influenza, to 1 in 81,818; 1 in 6666 of rheumatics, to 1 in 18,544.

New York has a greater mortality than London in diphtheria, croup, typhus and typhoid and puerperal fever, dysentery, diarrhœa, cholera, and ague. Thus, only 1 in 3755 of population die of diphtheria in London, while as many as 1 in 918 die in New York; 1 in 3111 of croup, to 1 in 901; 1 in 1032 of typhus and typhoid fevers, to 1 in 854; 1 in 13,181 of puerperal fever, to 1 in 10,742; only 1 in 26,851 of dysentery in London, to 1 in 3146 of population in New York; 1 in 1212 of diarrhœa, to 1 in 380; 1 in 18,230 of cholera, and 1 in 7429; 1 in 152,681 of ague, to 1 in 56,259. Of all other zymotic diseases, 1 in 116,000 of population die in london, 1 in 150,000 in New York.

In the tenement houses of New York every 6-story building averages 24 families, of five or more persons each. Each person has a little over 15 square feet of ground area, and 480 cubic feet of air space in the whole house. In the apartments the allowance of air space is only 317 cubic feet, and in the dormitories but 89 feet to each person. A full 1000 cubic feet of air space are required.

The air (if pure) which an adult healthy man breathes *in* contains only 0.4 per 1000 volumes of *carbonic acid;* while that he breathes *out* contains 40 volumes per 1000, in addition to *fetid organic matter* and water-vapor to saturation. It requires at least 2000 cubic feet per hour, of pure air, to keep the exposed carbonic acid at 0.5 or 0.6 per 1000 volumes, and to remove entirely the fetid smell of organic matter exposed, to say nothing of the filth and smell of persons, clothes, cooking and food-utensils, remains of food and offal generally.

The carbonic acid of respiration is equally diffused through the air of a room, and is very rapidly got rid of by opening windows. But neither the fetid or organic matter, nor the watery vapor, diffuse rapidly nor thoroughly.

At least 30 grains, and perhaps 240 grains, of organic matter are given off from the lungs and skin, and from 25 to 40 ounces of water in 24 hours. The organic matter is made up of small particles of epithelium and fatty matter detached from the skin, and partly of an organic vapor given off from the lungs and mouth. It has a fetid smell, and is retained in a room for a long time, sometimes for 4 hours, even when there is free ventilation, showing that it is oxidized very slowly. It is absorbed most by wool, feathers, damphrally and moist paper ; and least by straw and horsehair. It is molecular, and floats in clouds in the air, when the odor of it is not always equally diffused through a room. A large quantity of carbonic acid, derived from respiration, always indicates a large quantity of organic matter, the smell of which generally becomes perceptible when the carbonic acid reaches 0.7 per 1000 volumes ; and is very strong when it amounts to 1 per 1000.

Besides the gaseous products strictly derived from the lungs, the air of most dwelling-houses, when examined by the æroscope, is found to contain many *epithelium cells ;* most of which are evidently derived from the skin. They are rubbed off and then float through the air, and often become the carriers of the contagion of scarlet fever and measles, as they are saturated with poison when these diseases prevail. The epithelium of the mouth, throat, and nostrils, are foliated, and in diphtheria, typhus and typhoid fevers, and thus load the air with poisonous particles.

In all tainted atmospheres of this kind, it seems that the germs of infusoria abound to a much greater extent than in pure air. The possibility of a direct transference from body to body of cells (or epithelium) undergoing special changes, is thus placed beyond doubt, and the doctrine of contagion receives an additional elucidation. It remains to be seen whether pus or epithelium cells, becoming dried in the atmosphere, can again, on exposure, become revivified. Some protophytes, like the prolococcus pluvialis, may be dried, and yet retain their vitality for years, and may be flown about in atmospheric currents.

The effect of the fetid air containing organic matter, except of carbonic acid and water, is very marked on many people, causing heaviness, headache, inertness, nausea, or even decided symptoms, such as heat of skin, quick pulse, furred tongue, loss of appetite, and thirst, lasting for 24 or over 48 hours.

Usually, the persons who are compelled to breath such an atmosphere are, at the same time, sedentary, and remain in a constrained position for many hours,, are also underfed, and perhaps intemperate. They soon become pale, lose their appetite, decline in muscular strength and spirits. They are very apt to become scrofulous and consumptive. Baudelocque long ago asserted that impure air is the great cause of scofula, and that hereditary predisposition, syphilis, uncleanliness, want of clothing, bad food, and humid air, are, by themselves, non-effective. In the Dublin House of Industry, where consumption was so common as to be thought contagious, there were in one ward, 60 feet long and 18 broad, 38 beds, each containing four children; the atmosphere was so bad that, in the morning, the air of the ward was unendurable. The food was excellent, and the only causes for the excessive prevalency of consumption were foul air and want of exercise. In the prison of Leopoldstadt of Vienna, which was very badly ventilated, 378 prisoners died out of 4280, or one in twelve: and of these, no less than 220, or nearly two-thirds, died of consumption. There were no less than 42 cases of acute military tuberculosis. In the well-ventilated houses of correction, in Vienna, only 43 died out of 3037, or 1 in 71 : and of these only 24, or 1 in 126, died of phthisis. (But consumption is only a personal and family affection ; it is not handed from mouth to mouth, or from person to person, and thus made to invade the whole community.) The most important class of diseases produced by impurities in the atmosphere are certainly caused by the presence of organic matter floating in the air; and thence come all specific and contagious diseases. This organic matter may be present in the form of impalpable particles, or of moist or dried epithelium and pus-cells. It may be contained in the substances discharged or thrown off from the body, as in the discharges from the nose, throat, and lungs, of measles, scarlet fever, diphtheria, and whooping-cough patients; or in the epidermic scales of measles or scarlet-fever or erysipelas, or in the crusty scabs and pus of small-pox; or in the putrefactive changes in the discharges of typhus fever, cholera, dysentery, etc. And from the ease with which, in many cases, organic matters are absorbed by hydroscopic substances, it would appear that they may often be combined with, or condensed in, the water of the atmosphere.

The specific poisons differ greatly in the ease with which they are oxidized and destroyed. Thus, the poison of typhus exanthematicus is very easily got rid of by free ventilation, by means of which it is diluted and oxidized, so that it becomes inocuous at the distance of a few feet. (But if the streets, gutters, and sewers of houses in which typhus prevails are loaded with filth, and the scanty back yards are defiled by offensive cesspools and privies, free ventilation with pure air is impossible; then the volatile poison of typhus fever may unite itself with the impurities of the atmosphere, and perhaps convert the whole into a virulent miasma.) This is also the case with the poison of Oriental plague. But the poisons of small pox and scarlet fever will spread in spite of very free ventilation, and they retain their power of causing the same disease for a long time, and, in the case of scarlet fever, for months. (Then the scabs and epidermic scales are doubtless the active agents of propagation. In the one case, the poison may be a mere cloud of molecules; in the other it may be contained in the epithelium and pus-cells, thrown off from the skin in both cases, and from the throat also in one, which adhere to walls, clothing, or carpets, bscomes partially dry ; but then, becoming dislodged by sweeping, dusting,etc, etc., are blown up into the air and inhaled into the lungs of some one, where they again become active by means of warmth and moisture. Thus, scarlet fever, measles, small-pox, diphtheria, whooping-cough, typhus fever, etc., come up from the tenement houses and filthy parts of the cities, and are distributed to the well-to-do and wealthy. Convalescent small-pox and varioloid patients return to their work with their hair filled with crusts and scabs, and their clothes defiled with dried pus.

Scarlet fever and measles convalescents visit the houses of their patrons and friends with their unwashed heads filled with the scurf of measles and scarlet fever scales, and scatter it broadcast into the air, from whence it is inhaled into the nostrils, throat, or lungs, of some unsuspecting creature. They come also with their clothes contaminated with the dried expectoration of their children suffering with diphtheria and whooping-cough, and shake the dust of these poisons in the houses of the rich and philanthopic. Weavers, lace and ribbon makers, just recovering from small-pox, contaminate the new goods they manufacture, and dirty bank bills are often smeared with the same dangerous elements.—Ed. —*New York Med. Gazette.*

